# Remaining capacity estimation of lithium-ion batteries based on the constant voltage charging profile

**DOI:** 10.1371/journal.pone.0200169

**Published:** 2018-07-06

**Authors:** Zengkai Wang, Shengkui Zeng, Jianbin Guo, Taichun Qin

**Affiliations:** 1 School of Reliability and Systems Engineering, Beihang University, Beijing, China; 2 Science and Technology on Reliability and Environmental Engineering Laboratory, Beijing, China; Chongqing University, CHINA

## Abstract

Estimation of remaining capacity is essential for ensuring the safety and reliability of lithium-ion batteries. In actual operation, batteries are seldom fully discharged. For a constant current-constant voltage charging mode, the incomplete discharging process affects not only the initial state but also processed variables of the subsequent charging profile, thereby mainly limiting the applications of many feature-based capacity estimation methods which rely on a whole cycling process. Since the charging information of the constant voltage profile can be completely saved whether the battery is fully discharged or not, a geometrical feature of the constant voltage charging profile is extracted to be a new aging feature of lithium-ion batteries under the incomplete discharging situation in this work. By introducing the quantum computing theory into the classical machine learning technique, an integrated quantum particle swarm optimization–based support vector regression estimation framework, as well as its application to characterize the relationship between extracted feature and battery remaining capacity, are presented and illustrated in detail. With the lithium-ion battery data provided by NASA, experiment and comparison results demonstrate the effectiveness, accuracy, and superiority of the proposed battery capacity estimation framework for the not entirely discharged condition.

## 1. Introduction

Owing to the remarkable advantages of high energy density, environmentally friendly features, low self-discharge rate and long service life, lithium-ion batteries have been broadly used in various applications, such as hybrid electric vehicles (HEVs), electric vehicles (EVs) and consumer electronics [1, 2]. As the central power components, lithium-ion batteries should function stably to ensure the reliability and safety of the whole electric system. However, their performance inevitably deteriorates with cyclic usage. Once lithium-ion batteries degrade below their required working level, they can no longer perform their intended functions and may bring about extra maintenance costs, severe safety risks, or even irreparable catastrophic consequences [3].

To prevent possible accidents and help users make maintenance policies before batteries reach hazard levels, it is essential for the battery management system (BMS) to estimate the state of charge (SOC) and the state of health (SOH) of lithium-ion batteries. SOC is defined as the percentage of the battery remaining charge to the current maximum capacity [4], SOH characterizes the health status of the battery that is often represented as capacity loss or power loss [5]. Recently, myriad reliable and accurate approaches to estimate SOC have been studied, such as Coulomb-counting method [6, 7], intelligence-based methods [8, 9] and model-based methods [10–12]. Nevertheless, the SOH estimation methods is still crucial and much more challenging problem.

Actual battery capacity is a significant health indicator (HI) for describing the aging status, and monitoring this parameter can be applied for the SOH estimation [13]. When the remaining capacity decreases to a given threshold known as the end of life (EOL), the lithium-ion battery is regarded as to be failed. The battery capacity is defined as the maximum amount of electric charge that a fully charged battery can release, which can be calculated directly by measuring current under the controlled conditions. However, this direct computation method demands the battery to be fully discharged during operation, which is inefficient from an energy view [14]. Therefore, many approaches have been presented to estimate the battery capacity rather than perform the direct measurement, which can be further categorized into the model-based methods and the feature-based methods.

The model-based methods depend on an electrochemical model (EM), an equivalent circuit model (ECM) or an empirical model to describe the physical essence of the lithium-ion battery capacity degradation. The electrochemical model applies partial differential equations (PDEs) to describe the actual electrochemical reaction process inside batteries, which can capture dynamic behaviors with high accuracy [10]. For example, a pseudo two-dimensional (P2D) EM considering the temperature and porosity effects is proposed to estimate the capacity fading under the cyclic usage condition in Ref. [15]. Zheng et al. [10] proposed trinal proportional-integral (PI) observers with a one-dimensional spatial EM to simultaneously estimate SOC, capacity and resistance for lithium-ion batteries. Except for using an EM, some reseachers prefer to replace the battery with an ECM to estimate the battery SOC, thus the battery capacity can be obtained by computing the ratio of the time integral of the current to the difference value of the SOC for the same period. Wang et al. [2] integrated an n-order ECM with a sliding window neutral network (NN) to construct a probability based adaptive remaining capacity estimator. In Ref. [16], a simplified linearized ECM is presented to depict the battery dynamic characteristics, and an online recursive least square (RLS) and unscented Kalman filter (UKF) are combined to determine the model parameters for the capacity estimation. Since the integer-order ECM are not capable of predicting battery dynamics in both time and frequency domains over the whole operating range, the fractional-order modeling (FOM) techniques have been investigated for the battery applications [17–19]. The FOM can not only improve the estimation accuracy but also preserve some physical meanings underlying the model parameters. Zhang et al. [17] introduced the fractional-order ECM and corresponding fractional Kalman filter algorithm into the SOC estimation, and proved that their proposed SOC estimator can precisely track the true SOC trajectory in dynamic driving-cycle tests. Moreover, there are also some scholars selecting an empirical model to simulate the trend of capacity degradation. Saha et al. [20] proposed an exponential model according to the regression analysis of experimental data. Based on this exponential model, a particle learning framework with kernel smoothing is presented in Ref. [21]. Nevertheless, the disadvantages of the model-based methods are also evident: the electrochemical mechanism is too complicated to identify, the measurements of some parameters (such as open circuit voltage) involved in ECM require a very long rest time, and the estimation results based on those models usually come with large errors.

In contrast, the feature-based methods have a strong realistic significance. Since the battery capacity is related with several easily measured features, it is convenient to estimate capacity by using trained connections with multiple features from the current, voltage and temperature profiles. Those methods avoid understanding complex reaction mechanisms inside batteries to construct mathematical or physical models, thereby having been widely investigated by many researchers. For instance, Li et al. [22] extracted four characteristic parameters from charging voltage curves and constructed a particle filter (PF) model to estimate discharge capacity. Cheng et al. [23] applied visual cognition technique to build the capacity degradation model based on several geometrical features extracted from the current and voltage curves. To effectively capture the nonlinearity relationship between the features and capacity, various machine learning approaches have been integrated with feature-based methods as well. Inspired by the philosophy of human health and athletic ability estimation, Wu et al. [24] chose four differential geometric features from a specific charging sub-process to depict the battery health and modeled the battery capacity based on the group method of data handling (GMDH) polynomial neural network. Hu et al. [25] introduced the particle swarm optimization (PSO) to the k-nearest neighbor regression and built a data-driven estimation model based on five characteristics extracted from the constant current-constant voltage (CC-CV) charging process. The prognostic results show that these integrated methods often have a good performance in the battery capacity estimation.

Although the above feature-based methods can achieve relatively satisfactory estimation results, they require batteries should be fully discharged and charged, which can hardly be applicable in real operation. The discharging profiles of each cycle of batteries used in various conditions are diverse. During everyday usage, batteries are seldom fully discharged to the 0% SOC level but are usually recharged from a partially discharged state to the 100% SOC level. This incomplete discharging process will affect the initial state (such as the initial voltage) and processed variables (such as the charging time) of the subsequent charging process, thereby restricting the extraction of external features depending on a deterministic and intact charging/discharging process, such as the time ratio of CC phase to CV phase [22], image information transformed from the entire discharging data [23], the CC charging duration or capacity [25–27], discharging cutoff voltage [27], voltage variation in the CC phase [22, 24, 28], and time interval between two predifined discharging voltage [29], etc. Unfortunately, to the best of our knowledge, little work has been performed to solve this problem.

In fact, with the popular CC-CV charging mode, the CV profile is relatively robust with the incomplete discharging process and unstable initial charging state, and the BMS can reserve the corresponding charging data integrallty. Since the aim is to realize a credible online estimation of battery capacity whether the battery is fully discharged or not, therefore, illuminated by the data integrity and gradually aging phenomena in the CV profile, we extract a typical geometrical feature from the CV profile to be the aging indicator of battery capacity in this paper.

Based on the extracted aging feature, a data-driven remaining capacity estimation model is established by using a classical machining learning technique named support vector regression (SVR). SVR can deal with the nonlinear systems and outperform ordinary regression methods due to its robustness to small variations, excellent generalization capability and not directly affected by the dimension of regressed entities. Accordingly, SVR is often adopted as the learning model in studies of lithium batteries [30–32]. Since the performance of SVR highly relies on the selection of model parameters especially the kernel parameters, many intelligent algorithms like genetic algorithm (GA) [33, 34] and PSO [9, 35] are used to optimize the SVR model. Compared with GA, the PSO has faster convergence speed [36]. Nevertheless, the PSO also have some deficiencies including that its global convergence is not strictly guaranteed and it is easily trapped in the local optimal region [37]. Recently, enlightened by the concept from quantum mechanics, a new improved PSO algorithm named quantum particle swarm optimization (QPSO) has been developed by scholars [38, 39]. Different with the standard PSO, QPSO considers the state of each particle with a wave function instead of its velocity and position, thus it can theoretically guarantee to find the global optimal solution. Besides, it is only one parameter that needs to be controlled in QPSO, which makes QPSO easier to implement than PSO [40]. Motivated by this idea, this article proposes a novel remaining capacity estimation framework based on the quantum particle swarm optimization–based support vector regression (QPSO-SVR). The proposed approach extracted the aging feature from the CV profile and then estimate capacity based on the QPSO-SVR model. By using the data repository provided by NASA Ames Prognostics Center of Excellence (PCoE) [41], the advantages of our work in the battery capacity estimation over other benchmark approaches are fully demonstrated.

The structure of this paper is organized as follows: In section 2, the capacity degradation property of the lithium-ion battery is illustrated, and the aging feature from the CV charging profile for the capacity estimation is extracted. Section 3 briefly introduces the related estimation model and intelligent algorithm including the SVR model and the QPSO algorithm. The proposed capacity estimation framework based on the QPSO-SVR is described in detail in section 4. Experiment results are presented and analyzed in section 5. Finally, the conclusions are discussed in section 6.

## 2. Feature extraction based on constant voltage charging phase

### 2.1 Capacity degradation property

Since the monitoring impedance is too complicated in real application, the battery capacity is often employed as the indicator to reflect actual health condition of the lithium-ion battery. The capacity represents the total available charge that the lithium-ion battery can supply over time, which can be expressed as:

$$Q=\int_{t_{0}}^{t_{end}}I(t)dt$$ (1)

where *t*_0_ and *t*_*end*_ are the begin and end time of a charging/discharging cycle, *I*(*t*) denotes the charging/discharging current. Particularly, the capacity researched in this paper refers to the charging capacity.

**Fig 1:**
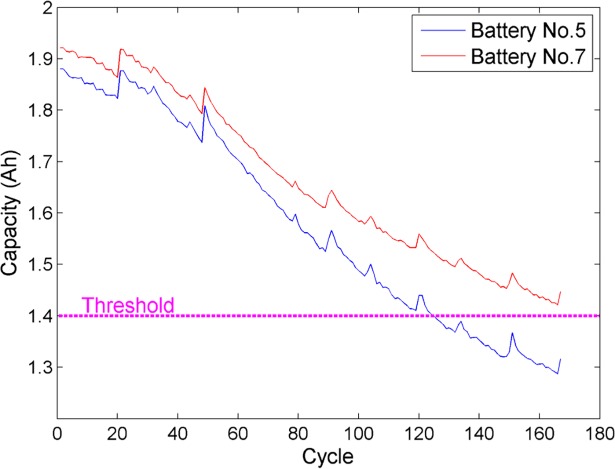
Measured charging capacity of lithium-ion batteries.

The remaining capacity of a lithium-ion battery is affected by many factors, such as external environmental loads, the number of charging and discharging cycles, the value of discharging current and so on. With the battery cycling, the capacity tends to be lower than the initial nominal value due to the loss of cyclic lithium and loss of active materials. Based on the data repository measured by NASA Ames PCoE, the charging capacity curves of battery No.5 and No.7 under nominal conditions are shown in Fig 1. Although the discharging levels of two batteries are different and there are some local fluctuations in capacity curves, it can be clearly observed that every capacity trajectory descends as cycle number increases, which corresponds with the fact that the battery SOH deteriorates gradually over time. Thus, the battery capacity is treated as the estimated objective in this paper.

### 2.2 Aging feature extraction

**Fig 2:**
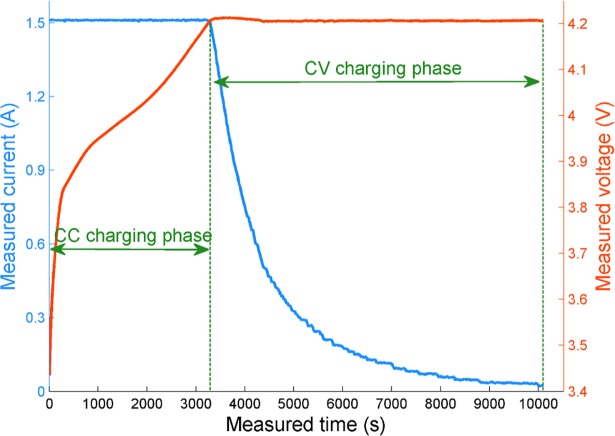
A typical CC-CV charging curve of the lithium-ion battery.

In actual operation, the partial discharging process not only limits the applications of some useful aging features which rely on an intact discharging process, but also does have underlying influences on the subsequent charging process. In order to avoid the negative effects caused by the incomplete discharging process as possible, the aging feature of the lithium-ion battery is extracted from the more controllable charging process in this work. The charging mode to be investigated in this paper is the CC-CV charging mode. As the most popular charging mode of lithium-ion batteries, the CC-CV protocol can be divided into two consecutive processes: the CC charging and the CV charging. As shown in Fig 2 (Battery No.5 from NASA Ames PCoE), during the CC profile, a varistor is exploited to keep the charging current constant until the battery voltage reaches the predefined maximum threshold. After that, the charging process turns into the CV profile and a constant voltage is imposed on the battery until the charging current drops to the predefined minimum threshold.

For the CC-CV mode, it is worth pointing out that only the CC profile is affected by the previous discharging state. Since if the battery goes through an incomplete discharging process, namely, the battery is not discharged to the predetermined cut-off voltage value, thus the initial battery voltage of the subsequent charging step will be higher than usual. As a result, the charging time will be undoubtedly decreased and the battery voltage trajectory will become uncertain, thereby making the CC profile not suitable for the aging feature extraction in practice. By contrast, as the battery turns into the CV phase when the voltage reaches the predefined maximum, it is evident that the CV profile is comparably robust with the unpredictable initial charging voltage. In other words, despite whether the battery is fully discharged or not, the process information can be completely saved and recorded in the dynamic data of the CV profile. Consequently, the CV profile is appropriate for the aging feature extraction under the condition of incomplete discharging.

**Fig 3:**
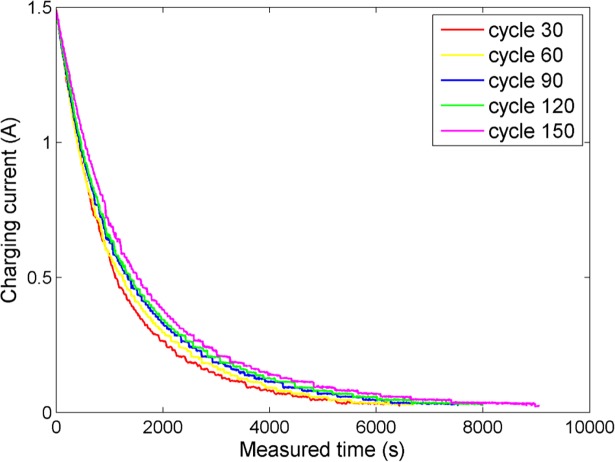
Aging phenomena during the CV profile at several cycles.

The more visualized aging phenomena during the CV profile can be explained by plotting the external measuring data of the lithium-ion battery. Fig 3 shows the CV charging current time series under different charging cycles (Battery No.5 from NASA Ames PCoE). It can be seen that with the cycle number increasing, the battery health condition becomes worse, thus the charging curves’ shape during the CV profile also changes distinctly and regularly. These gradually changing phenomena mainly derive from the loss of lithium inventory (LLI). LLI is one of the significant reasons leading to the capacity degradation of the lithium-ion battery. More importantly, according to the research given by Ref. [42], 5.5% of LLI occurs in the CC phase, while 94.5% takes place in the CV phase. That is, LLI is more evident to be observed during the CV profile. Therefore, it can be said that the gradually changed characteristic extracted from the CV charging can effectively describe the variation of battery capacity. Since the area under the CV charging current curve becomes larger apparently as the charging cycle passes, this study extracts the geometrical area under the CV charging current curve (the CV charging capacity) to be the battery aging feature for the not fully discharged situation. The CV charging capacity can be calculated by:

$$Q_{CV}=\int_{t_{CV}}^{t_{end}}I(t)dt$$ (2)

where *t*_*CV*_ and *t*_*end*_ are the begin and end time of the CV profile, *I*(*t*) denotes the time-varying charging current. Based on the aging feature extracted from the CV profile, the next step is to build an accurate and reliable model for the battery capacity estimation.

## 3. Related work

### 3.1 Support vector regression

SVR is a famous and powerful machine learning technique for both linear and nonlinear data regression. The basic SVR is formulated as convex quadratic programming (QP) problem which can condense the substantial training data into a dramatically smaller set of support vectors (SVs). Consider a dataset ${\left\{x_{i},y_{i}\right\}}_{i=1}^{N}$ ($x_{i}\in {\mathbb{R}}^{d}$ and $y_{i}\in \mathbb{R}$), where **x**_*i*_ is the input vector, *y*_*i*_ is the real output value, and *N* is the number of data samples. The SVR function can be described as:

$$f(x)=w^{T}x+b$$ (3)

where *f*(*x*) denotes the output value of prediction, **W** $\left(w\in {\mathbb{R}}^{d}\right)$ and *b* (b∈ℝ) are adjustable coefficients computed by learning from the dataset ${\left\{x_{i},y_{i}\right\}}_{i=1}^{N}$.

Aiming at finding a proper function (Eq (3)) such that the maximum deviation of *f*(*x*) with the training dataset is less than the predefined error term *ε*, the estimated value of **W** and *b* can be obtained by solving the following QP problem:

$$\begin{array}{cc} \underset{w,b}{\min} & \frac{1}{2}{\left\|w\right\|}^{2} \end{array}$$ (4)

subject to

$$-\varepsilon \leq y_{i}-w^{T}x_{i}-b\leq \varepsilon ,i=1,2,\cdots ,N$$ (5)

For the cases that training samples in the dataset are not linearly separable, the relevant constraints are relaxed by introducing defined slack variables $\xi_{i}^{\left(*\right)}$ for each sample point. The QP problem of SVR can be rewritten as:

$$\begin{array}{cc} \underset{w,b}{\min} & \frac{1}{2}{\left\|w\right\|}^{2}+C\sum_{i=1}^{N}\left(\xi_{i}+\xi_{i}^{*}\right) \end{array}$$ (6)

subject to

$$\{\begin{array}{c} w^{T}x_{i}-b-y_{i}\leq \varepsilon +\xi_{i}\\ y_{i}-w^{T}x_{i}-b\leq \varepsilon +\xi_{i}^{*}\\ \xi_{i},\xi_{i}^{*}\geq 0,i=1,2,\cdots ,N \end{array}$$ (7)

where *C* is the penalty parameter which represents the degree of attention paid to the outliers. Generally, the larger *C* is, the more attention will be paid to the outliers.

To solve the above optimization problem, the Lagrangian is adopted here:

$$\begin{array}{l} L(w,b,\xi^{\left(*\right)},\alpha^{\left(*\right)},\eta^{\left(*\right)})=\frac{1}{2}{\left\|w\right\|}^{2}+C\sum_{i=1}^{N}\left(\xi_{i}+\xi_{i}^{*}\right)-\sum_{i=1}^{N}\left(\eta_{i}\xi_{i}+\eta_{i}^{*}\xi_{i}^{*}\right)\\ \;-\sum_{i=1}^{N}\alpha_{i}(\varepsilon +\xi_{i}+y_{i}-w^{T}x_{i}-b)-\sum_{i=1}^{N}\alpha_{i}^{*}(\varepsilon +\xi_{i}^{*}+y_{i}-w^{T}x_{i}-b) \end{array}$$ (8)

where $\alpha^{\left(*\right)}={\left(\alpha_{1},\alpha_{1}^{*},\cdots ,\alpha_{N},\alpha_{N}^{*}\right)}^{T}$ and $\eta^{\left(*\right)}={\left(\eta_{1},\eta_{1}^{*},\cdots ,\eta_{N},\eta_{N}^{*}\right)}^{T}$ are corresponding Lagrange multipliers.

Taking the partial derivative of *L*(**w**,*b*,*ξ*^(*)^,*α*^(*)^,*η*^(*)^) with respect to original variables and substituting the results into Eq (8), the final dual expression of the SVR is shown as:

$$\underset{\alpha^{\left(*\right)}\in {\mathbb{R}}^{2N}}{\min}\frac{1}{2}\sum_{i,j=1}^{N}\left(\alpha_{i}^{*}-\alpha_{i})(\alpha_{j}^{*}-\alpha_{j})(x_{i}\cdot x_{j}\right)+\varepsilon \sum_{i=1}^{N}\left(\alpha_{i}+\alpha_{i}^{*}\right)-\sum_{i=1}^{N}y_{i}(\alpha_{i}^{*}-\alpha_{i})$$ (9)

subject to

$$\{\begin{array}{c} \sum_{i=1}^{N}(\alpha_{i}^{*}-\alpha_{i})=0\\ 0\leq \alpha_{i}^{\left(*\right)}\leq C \end{array}$$ (10)

where (**x**_*i*_ ⋅ **x**_*j*_) denotes the dot-product of two input vectors. Hypothesizing that ${\bar{\alpha }}^{\left(*\right)}={\left({\bar{\alpha }}_{1},{\bar{\alpha }}_{1}^{*},\cdots ,{\bar{\alpha }}_{N},{\bar{\alpha }}_{N}^{*}\right)}^{T}$ is the optimum solution and $\bar{b}$ is corresponding estimated value based on ${\bar{\alpha }}^{\left(*\right)}$. For any test sample x*, the design function of the SVR can be then given by:

$$y=g(x)=\sum_{i=1}^{N}\left({\bar{\alpha }}_{i}^{*}-{\bar{\alpha }}_{i})(x_{i}\cdot x^{*}\right)+\bar{b}$$ (11)

However, many problems show nonlinear features in the actual application. To realize the linear regression, a mapping function is exploited to transfer the nonlinear data in the original dimension space to the high dimension space. In the high dimension space, the kernel function is adequate to replace the dot-product of two vectors as well as prevent the curse of dimensionality.

There are several types of the kernel function, such as radial basis function (RBF) kernel, polynomial kernel, sigmoid kernel and so on. Due to only one parameter to be set and excellent generalization ability, the RBF kernel is the most choice when facing with nonlinear regression [43]. Consequently, the RBF kernel is selected as the mapping function in this work:

$$K(x_{i},x_{j})=\exp(-\frac{1}{2}\cdot \frac{{\left\|x_{i}-x_{j}\right\|}^{2}}{\sigma^{2}})$$ (12)

where *σ* denotes the predefined bandwidth of the RBF kernel. Therefore, the final design function of the SVR can be rewritten as:

$$y=\sum_{i=1}^{N}\left({\bar{\alpha }}_{i}^{*}-{\bar{\alpha }}_{i})K(x_{i},x^{*}\right)+\bar{b}$$ (13)

### 3.2 Quantum particle swarm optimization

PSO is a heuristic swarm intelligent algorithm broadly adopted in solving the optimization problems. The main idea of PSO derives from the imitation of biological and sociological predation behavior of birds swarm: the behavior of each particle (candidate solution) is subject to the Newtonian dynamics. Thus the update of each particle in the search space can be decided by its velocity and position:

$$v_{i,j}^{k+1}=\omega v_{i}^{k}+c_{1}r_{1}(pbest_{i}^{k}-x_{i}^{k})+c_{2}r_{2}(gbest^{k}-x_{i}^{k})$$ (14)

$$x_{i}^{k+1}=x_{i}^{k}+v_{i}^{k+1}$$ (15)

where $v_{i}^{k}$ and $x_{i}^{k}$ represents the velocity and position of particle *i* at iteration *k*, $pbest_{i}^{k}$ denotes the individual optimal solution of the particle *i* after *k* iterations, *gbest*^*k*^ denotes the global optimal solution after *k* iterations, *ω* is the inertia weight coefficient, *r*_1_ and *r*_2_ are two random numbers sampled from [0,1], *c*_1_ and *c*_2_ are learning factors. However, the traditional PSO algorithm also confronts some intractable problems in actual applications: its global convergence is not guaranteed; it is easily trapped in the local optimal solution; the algorithm requires too many parameters which are hard to be predetermined.

To overcome these disadvantages of PSO, QPSO was developed by introducing the quantum mechanics into the convergence process of PSO. From the quantum mechanics perspective, the velocity and position of the particle cannot be determined simultaneously due to the famous uncertainty principle [23]. Therefore, each particle involved in QPSO is hypothesized in a quantum state and is characterized by a wave function rather than its velocity and position. Assume that each particle moves in quantum space and there is a center point vector *p*_*i*_ which is used to constrain the movement of the particle *i*, so the wave function *ψ* of the particle *i* can be depicted by:

$$\psi (x_{i})=\frac{1}{\sqrt{L_{i}}}\exp\left(-\frac{\left|p_{i}-x_{i}\right|}{L_{i}}\right)$$ (16)

where *x*_*i*_ is the position of particle *i*, *L*_*i*_ represents the Delta potential well. To address the possibility of a particle’s appearance in the quantum space, the probability density function (PDF) of the particle *i* is computed as:

$$f(x_{i})={\left|\psi (x_{i})\right|}^{2}=\frac{1}{L_{i}}\exp\left(-\frac{2\cdot \left|p_{i}-x_{i}\right|}{L_{i}}\right)$$ (17)

By applying the Monte Carlo method, the position of the particle *i* can be updated as follows:

$$x_{i}^{k+1}=\{\begin{array}{c} p_{i}^{k}+\frac{L_{i}^{k}}{2}\ln\left(1/u\right),if\;\beta \geq 0.5\\ p_{i}^{k}-\frac{L_{i}^{k}}{2}\ln\left(1/u\right),if\;\beta <0.5 \end{array}$$ (18)

where *u* and *β* are two random number distributed uniformly in [0,1], and the value of $L_{i}^{k}$ can be given by:

$$L_{i}^{k}=2\cdot \alpha \cdot \left|mbest^{k}-x_{i}^{k}\right|$$ (19)

where *α* is called the contraction-expansion coefficient which controls the convergence speed of the particle and is the only parameter needs to be determined in QPSO, $mbest_{j}^{k}$ represents the mean best position of all *M* particles at *k* iteration:

$$mbest^{k}=\frac{1}{M}\sum_{i=1}^{M}pbest_{i}^{k}$$ (20)

Finally, substitute Eq (19) into Eq (18), the iterative updating formula can be written as:

$$x_{i}^{k+1}=\{\begin{array}{c} p_{i}^{k}+\alpha \cdot \left|mbest^{k}-x_{i}^{k}\right|\cdot \ln\left(1/u\right),if\;\beta \geq 0.5\\ p_{i}^{k}-\alpha \cdot \left|mbest^{k}-x_{i}^{k}\right|\cdot \ln\left(1/u\right),if\;\beta <0.5 \end{array}$$ (21)

Additionally, considering the convergence of *x*_*i*_, $p_{i}^{k}$ is defined as:

$$p_{i}^{k}=\varphi \cdot pbest_{i}^{k}+(1-\varphi )\cdot gbest^{k}$$ (22)

where *φ* denotes a random number distributed uniformly in [0,1].

## 4. The proposed capacity estimation framework based on the QPSO-SVR

**Fig 4:**
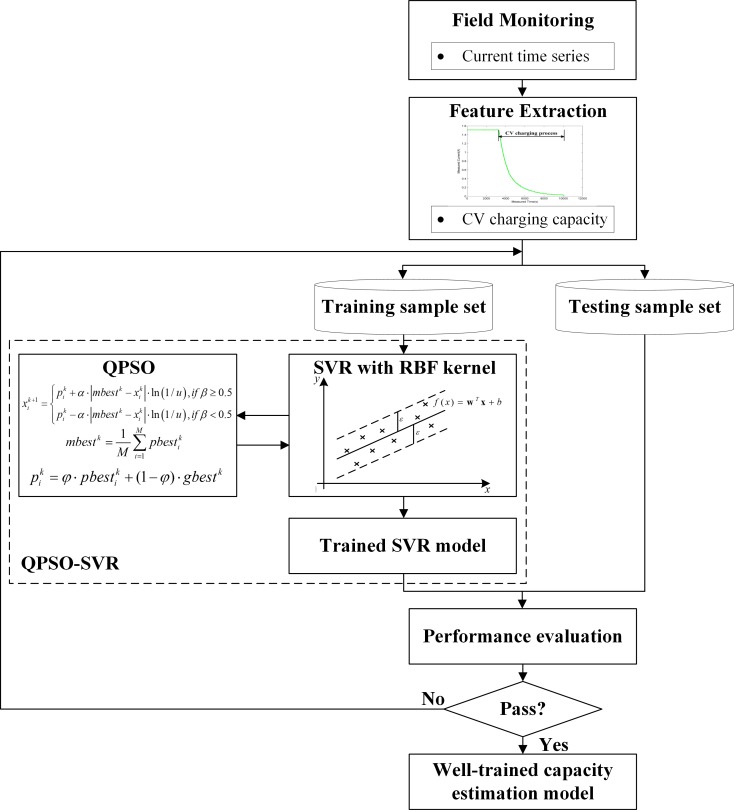
Schematic diagram of the proposed fusion framework for the lithium-ion battery capacity estimation.

Aiming to estimate the capacity of the lithium-ion battery by only using the charging data of the CV step, herein, we focus on how to construct an effective and accurate estimation model. By introducing the quantum computing theory into the classical machine learning technique, a fusion framework based on QPSO-SVR for the lithium-ion battery capacity estimation is proposed in this work. The schematic diagram of the proposed fusion framework is shown in Fig 4, and the main steps are summarized as follows:

### 4.1 Data collection

During the charging process of the lithium-ion battery, the monitored parameters including current, voltage, and temperature can be well recorded by a variety of sensors inside electrical appliances. The raw data required for the model construction is the charging current time series.

### 4.2 Feature extraction

Since the aging feature presented in this paper is extracted from the CV charging phase, the CV current time series should be separated from the whole charging dataset. The initial point of the CV step can be determined by recognizing whether the charging voltage reaches the predefined maximum value or not, while the endpoint can be detected by recognizing whether the load current falls to the predefined terminal value or not. Based on the separated data, the CV capacity series can be extracted by computing the area under the current curve. The extracted CV capacity and the total charging capacity are regarded as the input and output in the estimation model, respectively.

### 4.3 Model training

**Fig 5:**
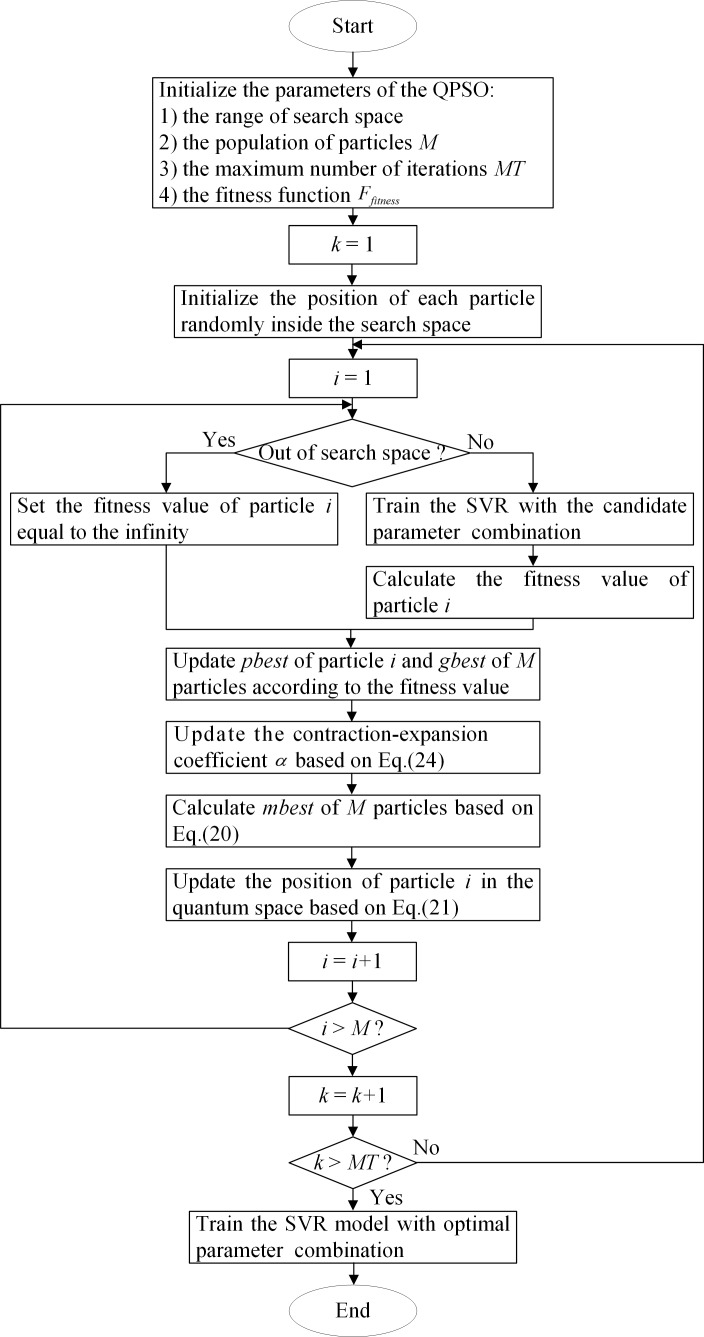
Flowchart of the presented QPSO-SVR method.

To ensure the generalizability of the estimation model, we divide the extracted sample sets into two parts: one part for the model training and the other part for the model testing. Training sample set is exploited to determine the optimal combination of the unknown parameters in the SVR model. To ensure the global optimization ability and improve the estimation accuracy of the SVR model, a novel QPSO-SVR method is presented, and the corresponding flowchart is shown as Fig 5 in detail.

The procedure of implementing the QPSO-SVR method is briefly described as below:

1. Define the search space of parameters to be optimized (including the penalty parameter *C*, the error term *ε*, and the bandwidth *σ*), the population of particles *M*, the maximum number of iterations *MT*, and the fitness function *F*_*fitness*_. It is worth pointing out that since the fitness function is used to judge the optimal position of particles in the quantum space, we select the mean square error (MSE) to be the fitness function, which can be given by:
  $$F_{fitness}=\frac{1}{N}\sum_{j=1}^{N}{\left(y_{j}-{\hat{y}}_{j}\right)}^{2}$$ (23)
  where *y*_*j*_ and ${\hat{y}}_{j}$, respectively, mean real value and training output value of *j*th sample in *N* training samples.
2. Initialize the position of each particle randomly inside the search space.
3. For the particle *i* (*i* = 1,2,⋯,*M*) appearing in the quantum space,
  - Decide whether the position of particle *i* is out of the search space or not. If its position is out of the search space, set the corresponding fitness value equal to the infinity; otherwise, substitute the candidate parameter combination into the SVR model and train the model with the training sample set, then calculate this particle’s fitness value based on Eq (23).
  - Update $pbest_{i}^{k}$ of particle and *gbest*^*k*^ of *M* particles according to the fitness value. Apparently, the less *F*_*fitness*_ is, the better position the particle has.
  - Calculate the value of the coefficient *α*. In this paper, *α* is updated adaptively as follows:
    $$\alpha =\alpha_{\max}-\frac{k}{MT}\left(\alpha_{\max}-\alpha_{\min}\right)$$ (24)
    where *α*_max_ and *α*_min_ are the predefined maximum and minimum of *α*, respectively.
  - Compute *mbest*^*k*^ of *M* particles based on Eq (20).
  - Update the position of particle *i* based on Eq (21).
4. Loop to 3) until the maximum iterations are reached.
5. By iteratively performing the above procedures, the optimal combination $\left(\hat{C},\hat{\varepsilon },\hat{\sigma }\right)$ can be found, and a trained SVR model can be then established.

### 4.4 Performance evaluation

Training sample set can be utilized to train the estimation model, but not to test the generalizability of the trained SVR. Consequently, we build a supervised learning step in this framework and some typical evaluation criteria are adopted to evaluate the performance of the trained model based on the testing sample set. The more details about this step can be found in section 5. According to the evaluation results, we can decide whether we need to retrain the estimation model or not.

Finally, based on the above framework, a well-trained capacity estimation model can be employed for lithium-ion batteries.

## 5. Experiment and analysis

### 5.1 Data source and correlation analysis

To make our work more detailed and comprehensible, experimental data from NASA battery dataset is used in this paper. In NASA accelerative aging test procedure, the commercial 18650 sized lithium-ion batteries (No.5 & No.7) went through three diverse operational profiles, namely, charging, discharging and impedance at room temperature. Charging was carried out in the CC mode at 1.5A until the battery voltage reached 4.2V. Afterward, batteries were continued charging in the CV mode until the charge current fell to 20mA. Discharging was conducted at the CC level of 2A until the battery voltage reduced to 2.7V and 2.2V for batteries No.5 and No.7, respectively. What’s more, 168 charging and discharging cycles were recorded.

**Fig 6:**
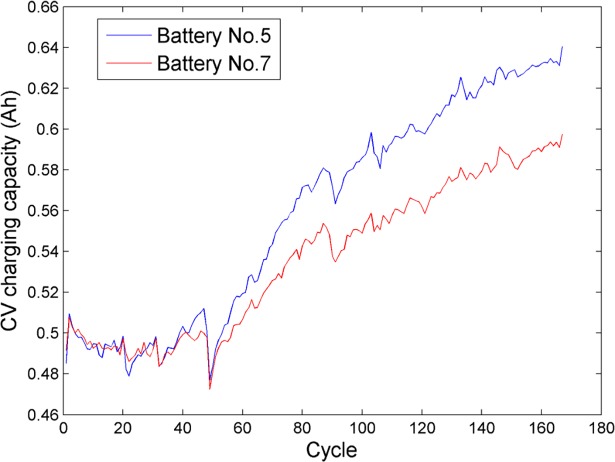
CV charging capacity of lithium-ion batteries.

By using the NASA lithium-ion battery datasets, aging features merely extracted from the CV step of battery No.5 and No.7 are shown in Fig 6. It can be seen that variation tendencies of two batteries are consistent, namely, at the beginning the CV capacity changes slightly as the cycle increases, then after approximately quarter of the cycles elapsing, the CV capacity starts to increase rapidly, which is consistent with the degradation trend of the capacity to some extent. In addition, as the battery capacity does not strictly degenerate in a monotonous way, the variation curve of the extracted feature also seems unsmooth for some cycles. All these facts show that there is a distinct underlying relationship between the battery capacity and the extracted aging feature.

To quantitatively demonstrate this underlying relationship, Pearson’s linear correlation coefficient and the Spearman’s rank correlation coefficient are calculated and analyzed, respectively.

The Pearson’s linear correlation coefficient is usually used to measure the linear dependence between two variables, which can be calculated as:

$$\rho =\mathrm{cov}(Q,Q_{CV})/\sigma_{Q}\sigma_{Q_{CV}}$$ (25)

where ***Q*** denotes the capacity vector with the standard deviation *σ*_*Q*_, ***Q***_*CV*_ denotes the extracted CV capacity vector with the standard deviation $\sigma_{Q_{CV}}$. The coefficient *ρ* ranges from −1 to 1, and the closer its absolute value near to 1, the stronger the linear dependence between two vectors.

Another statistical index is the Spearman’s rank correlation coefficient which is defined as the Pearson correlation between the rank values of two variables in descending order:

$$r=1-\frac{6}{n(n^{2}-1)}\sum_{i=1}^{n}d_{i}{}^{2}$$ (26)

where $d_{i}=Q_{i}^{\prime }-Q_{CVi}^{\prime }$, $Q^{\prime }=\{Q_{1}^{\prime },Q_{2}^{\prime },\cdots ,Q_{n}^{\prime }\}$ and $Q_{CV}^{\prime }=\{Q_{CV1}^{\prime },Q_{CV2}^{\prime },\cdots ,Q_{CVn}^{\prime }\}$ are the rearranged vectors of two series ***Q*** and ***Q***_*CV*_ in descending order. Similarly, the coefficient *r* also ranges from −1 to 1, and the closer its absolute value near to 1, the stronger the monotonic correlation between two vectors.

Based on the previous two indices, the results of correlation analysis are listed in Table 1. It can be seen that correlation coefficients *ρ* and *r* are very close to -1 for both battery No.5 and No.7, which imply a strong negative correlation between the battery capacity and the extracted aging feature. In other word, the quantitative correlation analysis verifies the feasibility of the building the capacity estimation model based on the extracted aging feature once again.

**Table 1:** Correlation analysis of the extracted aging feature and battery capacity.

Battery	*ρ*	*r*
**No.5**	−0.9914	−0.9784
**No.7**	−0.9806	−0.9644

### 5.2 Evaluation metrics for estimation performance

Before implementing the presented QPSO-SVR approach to estimate capacity of the lithium-ion battery, some metrics are necessary to evaluate the estimating performance and accuracy. Here we employ the mean absolute percentage error (MAPE) and root mean square error (RMSE) to act as the evaluation criteria of different methods:

$$MAPE=\frac{1}{N}\sum_{i=1}^{N}\left|\frac{y_{i}-{\hat{y}}_{i}}{y_{i}}\right|\times 100\%$$ (27)

$$RMSE=\sqrt{\frac{1}{N}\sum_{i=1}^{N}{\left(y_{i}-{\hat{y}}_{i}\right)}^{2}}$$ (28)

where *y*_*i*_ is the actual capacity value, ${\hat{y}}_{i}$ is the estimated capacity value and *N* is the sample size.

Since the lithium-ion battery is recognized as invalid when the charging capacity decreases to 70% or 80% of the rated capacity [44], we define 70% of the rated capacity (1.4 Ah) as the failure threshold in this work. However, it is noted that in Fig 1, the capacity of battery No.5 has crossed the failure threshold, while the capacity of battery No.7 has not dropped below the failure threshold even at the end of the experiment. Accordingly, this article also performs remaining useful life (RUL) estimation especially for battery No.5 to further verify the effectiveness of the proposed framework. Thus, the other two evaluation metrics including the absolute error *e*_*a*_ and the relative error *e*_*r*_ are exploited in RUL estimation:

$$e_{a}=\left|RUL_{real}-RUL_{estimated}\right|$$ (29)

$$e_{r}=\left|RUL_{real}-RUL_{estimated}\right|/RUL_{real}$$ (30)

### 5.3 Estimation results and analysis

The experimental datasets of battery No.5 and No.7 are used to evaluate our extracted aging feature and proposed remaining capacity estimation framework. Each dataset is separated into two parts: one part for the model construction and the other part for the capacity estimation. To demonstrate the superiority of the presented QPSO-SVR method, this paper compares the capacity estimation results obtained by the standard SVR (denoted as SVR) and the SVR optimized by PSO algorithm (denoted as PSO-SVR). Furthermore, considering the practical meanings for maintenance and logistics support of lithium-ion batteries, we also choose different charging cycles (the 80th cycle, the 90th cycle, and the 100th cycle) as the estimation starting point.

The whole experiments are operated on the MSI brand laptops by Intel Core i7 2.5 GHz CPU and 16 GB Memory. The software platform is the MATLAB R2014a.

**Fig 7:**
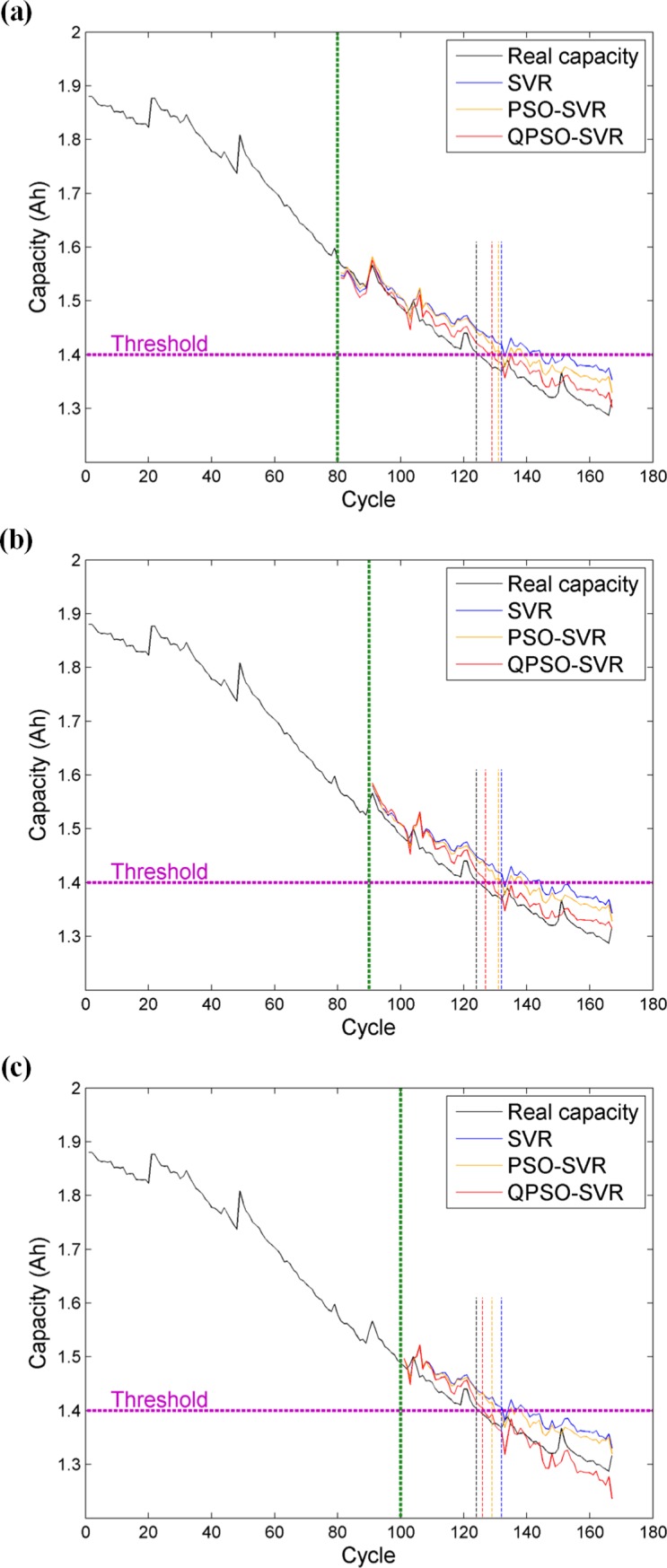
**Capacity and RUL estimation for battery No.5 at different starting points:** (a) at the 80th point; (b) at the 90th point; (c) at the 100th point.

For battery No.5, the estimation results of different methods at different starting points are shown in Fig 7. We use, respectively, the data from the first 80 cycles, the first 90 cycles, and the first 100 cycles to train the estimation model, and the rest of data to evaluate the model performance. The curves representing the future degradation trend acquired by SVR, PSO-SVR, and QPSO-SVR are plotted in each subfigure. On the one hand, it can be discovered that three methods can relatively satisfactorily predict the trends of the lithium-ion battery capacity degradation, which further validate that our extracted aging feature is suitable for reflecting the real battery capacity. On the other hand, under the same starting point, the remaining capacity curve estimated by QPSO-SVR is closer to the actual degradation curve, while the curves estimated either by SVR or by PSO-SVR exhibit more significant deviation from the real one, especially when the actual degradation curve approaches the failure threshold. That is, the QPSO-SVR can provide a more accurate estimated value in the battery RUL estimation as well. In brief, Fig 7 indicates that the aging feature extracted from the CV profile can produce comparatively desirable estimation results intuitively, and suggests a higher accuracy of our method for battery No.5.

Table 2 provides a quantitative evaluation of the capacity and RUL estimation results for battery No.5. We can undoubtedly find that the QPSO-SVR has the smallest RMSE and MAPE among all three methods, indicating that the QPSO-SVR excels in tracking the degradation path. RUL estimation results are listed in Table 2, too. They also suggest that the QPSO-SVR produces the most accurate RUL estimations among those methods. For example, when the starting point is selected as the 90th cycle, the real EOL is the 124th cycle and the real RUL is 34 cycles. Based on the QPSO-SVR, the estimated EOL and RUL are 127th cycle and 37 cycles, respectively, and the corresponding absolute error *e*_*a*_ is only three cycles, which is observably less than the value of *e*_*a*_ based on SVR (eight cycles) and PSO-SVR (seven cycles). The same conclusions can be drawn for the other two starting points, manifesting that the proposed method can be well applied to RUL estimation no matter when the estimation performs.

**Table 2:** Comparison of capacity and RUL estimation results among different methods (battery No.5).

Starting cycle	Method	EOL	RUL	*e*_*a*_	*e*_*r*_	RMSE	MAPE (%)
**80**	SVR	132	52	8	0.1818	0.0477	3.0397
	PSO-SVR	131	51	7	0.1591	0.0367	2.3446
	QPSO-SVR	129	49	5	0.1136	0.0218	1.4007
**90**	SVR	132	42	8	0.2353	0.0484	3.2597
	PSO-SVR	131	41	7	0.2059	0.0390	2.6029
	QPSO-SVR	127	37	3	0.0882	0.0238	1.5229
**100**	SVR	132	32	8	0.3333	0.0419	2.8769
	PSO-SVR	129	29	5	0.2083	0.0345	2.3560
	QPSO-SVR	126	26	2	0.0833	0.0258	1.5028

**Fig 8:**
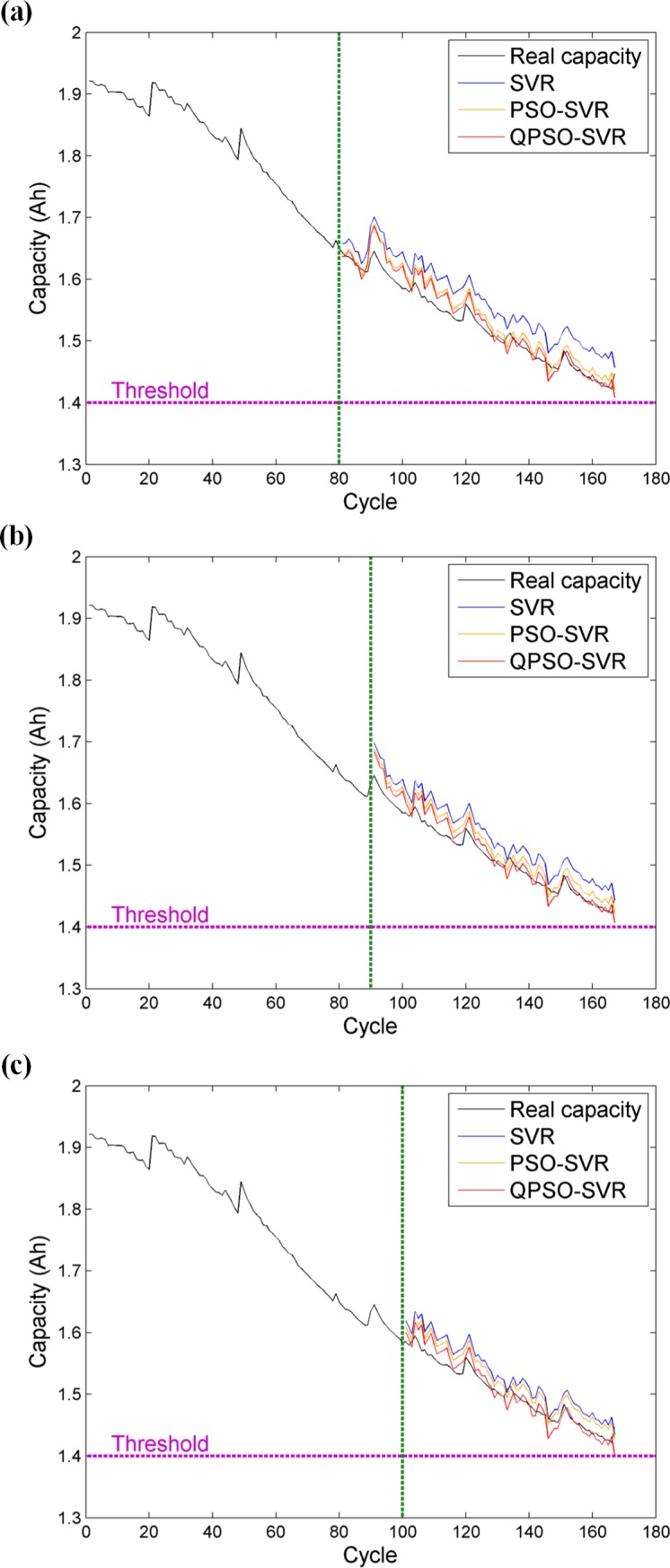
**Capacity estimation for battery No.7 at different starting points:** (a) at the 80th point; (b) at the 90th point; (c) at the 100th point.

Since the capacity of battery No.7 does not reach the threshold during the aging test, we only perform the capacity estimations at different starting points for battery No.7. The results are displayed in Fig 8 and Table 3. It is easily found that the intuitive results shown in Fig 8 basically comply with the analytical conclusions of battery No.5. We can see capacity curves estimated by our extracted feature have all grasped the global trend of actual capacity degradation, but the presented QPSO-SVR method significantly precedes the standard SVR and the PSO-SVR in estimation performance. From a quantitative perspective, the QPSO-SVR method also gives the most satisfactory estimation results for battery No.7. As listed in Table 3, the MAPEs of the QPSO-SVR at 80th, 90th and 100th are 0.9408, 0.9452 and 0.8083, respectively, which are all less than one percent and are the least values among the three comparative methods. Similarly, it is seen from Table 3 that the QPSO-SVR also has the smallest RMSE, demonstrating that the QPSO-SVR can yield more precise estimation than the SVR and the PSO-SVR.

**Table 3:** Comparison of capacity estimation results among different methods (battery No.7).

Starting cycle	Method	RMSE	MAPE (%)
**80**	SVR	0.0441	2.7754
	PSO-SVR	0.0223	1.2355
	QPSO-SVR	0.0185	0.9408
**90**	SVR	0.0378	2.3618
	PSO-SVR	0.0244	1.4109
	QPSO-SVR	0.0183	0.9452
**100**	SVR	0.0316	1.9555
	PSO-SVR	0.0236	1.4300
	QPSO-SVR	0.0159	0.8083

In conclusion, the abovementioned discussions prove that the geometrical feature extracted from the CV profile can be used to estimate the battery capacity, and the proposed QPSO-SVR approach observably outperforms the SVR and PSO-SVR approaches in a real application. By using the presented framework, we can obtain an accurate and credible capacity estimation result for the lithium-ion battery for the not entirely discharged condition.

## 6. Conclusions

To avoid being affected by the conventional incomplete discharging process of lithium-ion batteries, a novel data-driven framework is presented for the battery remaining capacity estimation. Since the CV charging profile is relatively robust with the unpredictable initial charging state, and the corresponding process information can be completely recorded, a geometrical feature of the CV charging curve, namely the CV charging capacity, is extracted to be the aging feature of lithium-ion batteries under the incomplete discharging condition. By introducing the quantum computing theory into the classical machine learning technique, a QPSO-SVR framework, as well as its application to the battery remaining capacity estimation problems, are presented and illustrated in detail.

With the data repository provided by NASA Ames PCoE, correlation analysis confirms that there is a distinct and strong relationship between the battery capacity and the extracted aging feature. The corresponding estimated remaining capacity further demonstrates the potential usefulness of the extracted CV capacity to be an independent aging feature for the not entirely discharged condition. Besides, compared with the conventional SVR and PSO-SVR under various evaluation metrics, the proposed QPSO-SVR framework also has the least estimation errors and shows stronger generalization performance in both capacity estimation and RUL estimation. Therefore, we can conclude that our extracted aging feature and the proposed framework are promising and credible for the remaining capacity estimation of lithium-ion batteries under the incomplete discharging process.
